# Assessing the occurrence and transfer dynamics of ESBL/pAmpC-producing *Escherichia coli* across the broiler production pyramid

**DOI:** 10.1371/journal.pone.0217174

**Published:** 2019-05-17

**Authors:** Ilias Apostolakos, Lapo Mughini-Gras, Luca Fasolato, Alessandra Piccirillo

**Affiliations:** 1 Department of Comparative Biomedicine and Food Science, University of Padua, Legnaro (PD), Italy; 2 Center for Infectious Disease Control, National Institute for Public Health and the Environment (RIVM), Bilthoven, the Netherlands; 3 Faculty of Veterinary Medicine, Utrecht University, Utrecht, the Netherlands; Panstwowy Instytut Weterynaryjny - Panstwowy Instytut Badawczy w Pulawach, POLAND

## Abstract

Extended-spectrum *β*-lactamase (ESBL)- and plasmid mediated AmpC-type cephalosporinase (pAmpC)-producing *Escherichia coli* (ESBL/pAmpC *E*. *coli*) in food-producing animals is a major public health concern. This study aimed at quantifying ESBL/pAmpC-*E*. *coli* occurrence and transfer in Italy’s broiler production pyramid. Three production chains of an integrated broiler company were investigated. Cloacal swabs were taken from parent stock chickens and offspring broiler flocks in four fattening farms per chain. Carcasses from sampled broiler flocks were collected at slaughterhouse. Samples were processed on selective media, and *E*. *coli* colonies were screened for ESBL/pAmpC production. ESBL/pAmpC genes and *E*. *coli* phylogroups were determined by PCR and sequencing. Average pairwise overlap of ESBL/pAmpC *E*. *coli* gene and phylogroup occurrences between subsequent production stages was estimated using the proportional similarity index, modelling uncertainty in a Monte Carlo simulation setting. In total, 820 samples were processed, from which 513 ESBL/pAmpC *E*. *coli* isolates were obtained. We found a high prevalence (92.5%, 95%CI 72.1–98.3%) in day-old parent stock chicks, in which *bla*_CMY-2_ predominated; prevalence then dropped to 20% (12.9–29.6%) at laying phase. In fattening broilers, prevalence was 69.2% (53.6–81.3%) at the start of production, 54.2% (38.9–68.6%) at slaughter time, and 61.3% (48.1–72.9%) in carcasses. Significantly decreasing and increasing trends for respectively *bla*_CMY-2_ and *bla*_CTX-M-1_ gene occurrences were found across subsequent production stages. ESBL/pAmpC *E*. *coli* genetic background appeared complex and *bla*-gene/phylogroup associations indicated clonal and horizontal transmission. Modelling revealed that the average transfer of ESBL/pAmpC *E*. *coli* genes between subsequent production stages was 47.7% (42.3–53.4%). We concluded that ESBL/pAmpC *E*. *coli* in the broiler production pyramid is prevalent, with substantial transfer between subsequent production levels.

## Introduction

Extended spectrum cephalosporins (ESCs) are considered critically important antimicrobials (CIAs) in both human and veterinary medicine [1,2]. Resistance to these antimicrobials is therefore a major public health concern due to the risks of therapy failure [3]. Resistance in *Enterobacteriaceae*, such as *Escherichia coli*, caused by production of extended-spectrum *β*-lactamases (ESBLs) or AmpC *β*-lactamases (pAmpCs) and transferability of resistance mechanisms are of particular importance, as the encoding genes (hereafter ESBL/pAmpC genes) are often located in promiscuous plasmids [4]. This property of EBSL/pAmpC genes enables their exchange between bacteria, including pathogens, and favours transmission between animals and humans [5]. Usage of ESCs in poultry has been restricted since 2012 in the European Union (EU) [6] except for occasional use in (grand)parent hatcheries [7,8]. However, numerous studies have shown high prevalence rates of ESBL/AmpC-producing *E*. *coli* (ESBL/pAmpC *E*. *coli*) in broiler flocks across Europe [9–11], even in countries with low antimicrobial use in livestock [7,12]. The presence of ESBL/pAmpC *E*. *coli* has been evidenced at all levels of the broiler production pyramid from breeding farms [13], hatcheries [14], fattening farms [15], slaughterhouses [16] to retail meat [17]. Whether broiler meat contaminated with ESBL/pAmpC *E*. *coli* represents an important source of human infections is debatable [18]. For instance, a recent comparative risk assessment estimated the exposure to ESBL/pAmpC *E*. *coli* through consumption of chicken meat to be lower than beef and pork [19], whereas a meta-analysis identified poultry products as the most likely source of human ESC-resistant infections [20]. The zoonotic transmission potential has prompted many investigations, not only on the prevalence of ESBL/pAmpC *E*. *coli*, but also on their transmission routes along the broiler production pyramid. A recent review [21] summarised the transfer of ESBL/pAmpC-producing bacteria in four major pathways; vertical transmission from parent to offspring, transmission in the hatchery, horizontal transmission in fattening farms, and horizontal transmission between farms or from the environment. Additionally, the authors stressed the need for more quantitative data on these transmission pathways.

In Italy, data on both prevalence and transmission dynamics of EBSL/pAmpC genes in poultry are lacking. This study aimed to establish a baseline prevalence of ESBL/pAmpC *E*. *coli* in the broiler production pyramid and to characterise the genetic background of resistant isolates in order to assess their transfer between subsequent levels of the broiler production pyramid.

## Materials and methods

### Sample collection

Three production chains (chain A, B, and C) of an integrated broiler production company in Northern Italy (specifically in Veneto, Lombardy, and Friuli-Venezia Giulia regions) were monitored from January 2017 to January 2018. In each of the 40 sampling visits, faecal samples from 20 randomly selected healthy birds were taken by cloacal swabs in the farms, and 20 carcasses were collected at the slaughterhouse. For each chain, sample collection started at the top of the production pyramid by sampling day-old Parent Stock (PS) chicks, which were the progeny of the Grandparent Stock (GPS) flock located elsewhere. Imported day-old PS chicks were sampled upon arrival to the rearing farm within one hour from delivery. Due to time constraints, samples were not collected for PS chicks of chain B. The same flock of PS chickens was sampled again during the laying period (~30-weeks-old) at the production farm (PS breeders), in which they were moved at the age of 21-weeks. Sampled breeder flocks were not combined with other flocks at the production farms. The offspring of the sampled PS breeders were tracked and sampled in four commercial fattening farms per production chain at two time points; at the age of one-day-old (broiler chicks), and around 30^th^ day of age, within the last week prior slaughter (broilers) (S1 Table). Samples in each broiler farm were collected from the same poultry house, which contained only the progeny of the previously sampled PS breeders. Finally, 20 carcasses from the previously sampled broilers were collected at the slaughterhouse after the chilling process. All breeder flocks and all broiler flocks, except three of them, received antibiotic treatment, mainly amoxicillin for therapeutic reasons unrelated to this study (S1 Fig). Broilers from the three monitored chains were all processed in the same slaughterhouse in Veneto region. Cloacal swabs from birds were collected by veterinarians of the private company as part of the routine monitoring of the flock health status and conducted in compliance with good veterinary practices.

### Isolation and detection

Cloacal swabs were directly streaked on Eosin Methylene Blue agar (Microbiol, Italy) supplemented with 1mg/L cefotaxime (CTX-EMB) and incubated at 37±0.5 °C for 20±2h. Carcasses were analysed by both a qualitative and quantitative method. For the qualitative method, carcasses were rinsed with Buffer Peptone Water (BPW), rinsates were incubated (37±0.5 °C for 20±2h) and streaked on CTX-EMB. For the quantitative method, rinsates and three serial dilutions (10^−1^ to 10^−3^) were plated on CTX-EMB for subsequent enumeration. One to two morphologically typical *E*. *coli* colonies on CTX-EMB (metallic green sheen) were isolated from each sample and subjected to species confirmation by combination of indole test and PCRs targeting *E*. *coli* housekeeping genes [22]. Confirmed *E*. *coli* isolates were screened for ESBL/pAmpC production by double-disk synergy test using cefotaxime (30 μg) and ceftazidime (30 μg) discs with and without clavulanic acid (10 μg) and according to CLSI guidelines [23]. Additionally, a cefoxitin disc (30 μg) was used to detect potential AmpC-producers.

### Molecular characterisation

ESBL/pAmpC gene groups [24] and *E*. *coli* phylogroups [25] were detected by multiplex PCRs for all phenotypically resistant isolates. For a selection of 119 isolates, ESBL/AmpC genes were sequenced (Macrogen, Spain) after amplification with the primers described by Dierikx *et al*. [26] to identify gene variants. This selection was done considering the variability of ESBL/pAmpC genes and *E*. *coli* groups per sampling (at least one isolate per phylogroup-*bla* gene combination per sampling). Moreover, isolates with an AmpC phenotype, but negative for pAmpC genes by multiplex PCR, were analysed for chromosomal mutations in the *ampC* promoter/attenuator (cAmpC) according to Haldorsen *et al*. [27].

### Data analysis

Prevalence of ESBL/pAmpC *E*. *coli* for each production stage was calculated based upon faecal samples or carcasses being positive to ESBL/pAmpC *E*. *coli* (at least one isolate). Prevalence estimates and corresponding 95% confidence intervals (95% CI) were adjusted for clustering of observations at the chain and farm levels using cluster-robust standard errors. Chi-square statistic for trends in proportions was used to test the significance of trends in the relative frequencies of different ESBL/pAmpC gene groups, gene variants, and phylogroups over subsequent sampling stages across the whole broiler production pyramid. Statistical analysis was performed using STATA (StataCorp, College Station, USA).

To quantify the possible transfer of ESBL/pAmpC *E*. *coli* across the broiler production pyramid, the average pairwise overlap of ESBL/pAmpC genes and *E*. *coli* gene groups (M1), gene groups plus gene variants (M2), or gene groups plus gene variants plus phylogroups (M3), between subsequent production stages, was estimated based on the proportional similarity index (PSI) [28], accounting for uncertainty in measurements in a Monte Carlo simulation setting. PSI values range from 0 (no similarities) to 1 (total overlap). The PSI is expressed as:

$$PSI=\left(1-0.5\sum |r_{j,x,y,k}-r_{j,x,y,k+1}|\right)$$

where *r* denoted the relative frequency of ESBL/pAmpC gene group (M1), ESBL/pAmpC gene variant (as additional strata within gene group) (M2), or *E*. *coli* phylogroup (as additional strata within the gene variant, which in turn were strata of the respective gene group) (M3) *j* (with *j* = 1,…, *J*; and *J* = 7 gene groups, *J* = 10 gene groups plus gene variants, and *J* = 7 gene groups plus gene variants plus phylogroups), in farm *x* (with *x* = 1,…, *X*; and *X* = 4) of chain *y* (with *y* = 1,…, *Y*; and *Y* = 3) at sampling stage (*i*.*e*. production level) *k* (with *k* = 1,…, *K*; and *K* = 5). The average of the PSIs calculated over the three chains and four farms per chain gave the overall measure of overlap between sampling stages, as follows:

$$\frac{1}{n}\sum_{i=1}^{n}{PSI}_{i}=\frac{{PSI}_{1}+{PSI}_{2}+\ldots +{PSI}_{n}}{n}$$

Uncertainty was introduced in the frequencies of M1, M2 and M3 by assuming the following probability distribution:

$$\left(r_{1,x,y,k},r_{2,x,y,k},\ldots ,1-\sum_{j=1}^{J-1}r_{j,x,y,k}\right)\approx Dirichlet(X_{1,x,y,k},X_{2,x,y,k},\ldots ,X_{J,x,y,k})$$

Where *r* is defined as above, and *X* is the number of detections of ESBL/pAmpC gene group/gene variant/phylogroup *j* in farm *x* of chain *y* at sampling stage *k*.

This analysis was performed in @RISK (Palisade Corp., USA) by setting 10,000 iterations with the Latin hypercube sampling technique and a seed of 1.

## Results

### Prevalence of ESBL/pAmpC *E*. *coli*

**Fig 1:**
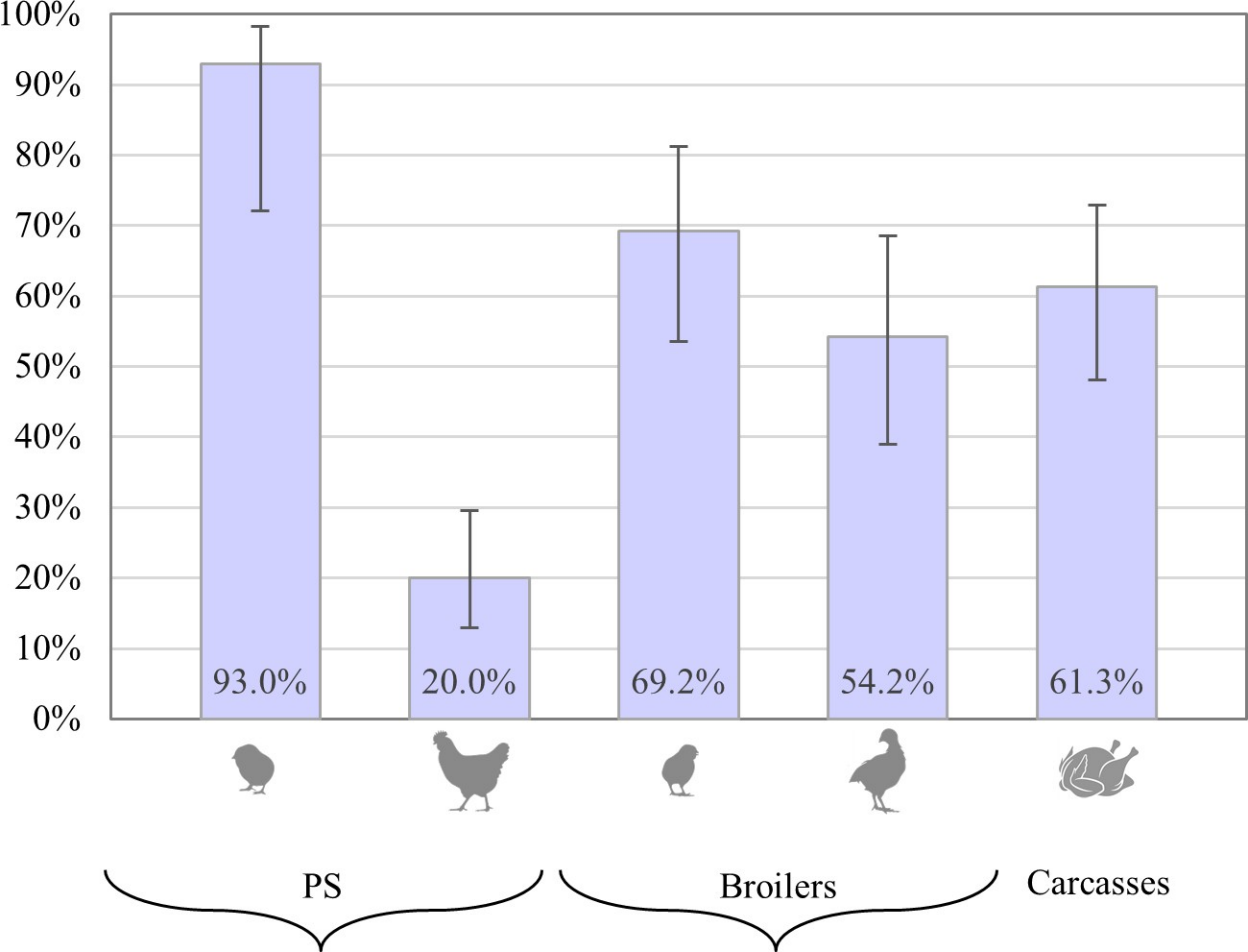
Prevalence of ESBL/pAmpC *E*. *coli* in the broiler production pyramid. From left to right: PS chicks, PS breeders, broiler chicks, broilers and carcasses. Bars represent corresponding 95% confidence intervals.

In total, 820 samples (*i*.*e*. 20 samples per chain-farm-sampling stage combination) were collected over 40 sampling visits. From these samples, 537 confirmed *E*. *coli* isolates from CTX-EMB were screened for phenotypic resistance to ESCs by disk diffusion and 513 (95.5%) thereof were positive. Overall, 60.3% (95%CI 51.3–68.1%) of samples were positive for presence of ESBL/pAmpC *E*. *coli* (Fig 1). ESBL/pAmpC *E*. *coli* were recovered at all sampling events on farms (cloacal swabs) and slaughterhouse (carcasses). A high prevalence (92.5%, 95%CI 72.1–98.3%) was found in day-old PS chicks, which dropped to 20% (95%CI, 12.9–29.6%) during the laying period. In fattening broilers, prevalence was higher again (69.2%, 95%CI 53.6–81.3%) at the start of the production cycle, and decreased to 54.2% (95%CI 38.9–68.6%) in the last sampling right before slaughter (Fig 1). At the end of the production pyramid, ESBL/pAmpC *E*. *coli* was isolated from 61.3% (95% CI 48.1–72.9%) of carcasses (Fig 1). In 29.2% of carcasses (*n* = 43 samples) positive for presence of ESBL/pAmpC *E*. *coli* by the qualitative method, the load was below the quantification limit (1 Log CFU/mL). In samples with countable ESBL/pAmpC *E*. *coli* the median load was 1.66 Log CFU/1 mL rinsing water (min 1 Log CFU/mL, max 4.2 Log CFU/mL).

### ESBL/pAmpC genes and phylogroup distribution

Out of the 513 ESC-resistant isolates, 333 (64.9%) had the ESBL and 180 (35.1%) the AmpC phenotype. By multiplex PCR, the following ESBL gene groups were identified: *bla*CTX-M-group-1 (41.9% of all resistant isolates), *bla*SHV (17.6%), *bla*CTX-M-group-9 (2.9%), *bla*CTX-M-group-2 (2.5%) and *bla*TEM (0.2%). All isolates with an AmpC phenotype were carrying *bla*CMY (25.6%), except for 18 isolates in which the following mutations were discovered in the *ampC* gene control region: −88 (C→T), −82 (A→G), −42 (C→T), −18 (G→A), −1 (C→T) and +58 (C→T). No ESBL/pAmpC genes were found for 28 ESC-resistant *E*. *coli*. One-hundred-eighty-three isolates with a confirmed ESBL/pAmpC gene had concurrent presence of *bla*TEM, which upon sequencing (*n* = 19 isolates) proved to be *bla*_TEM-1b_, a broad-spectrum *β*-lactamase.

**Fig 2:**
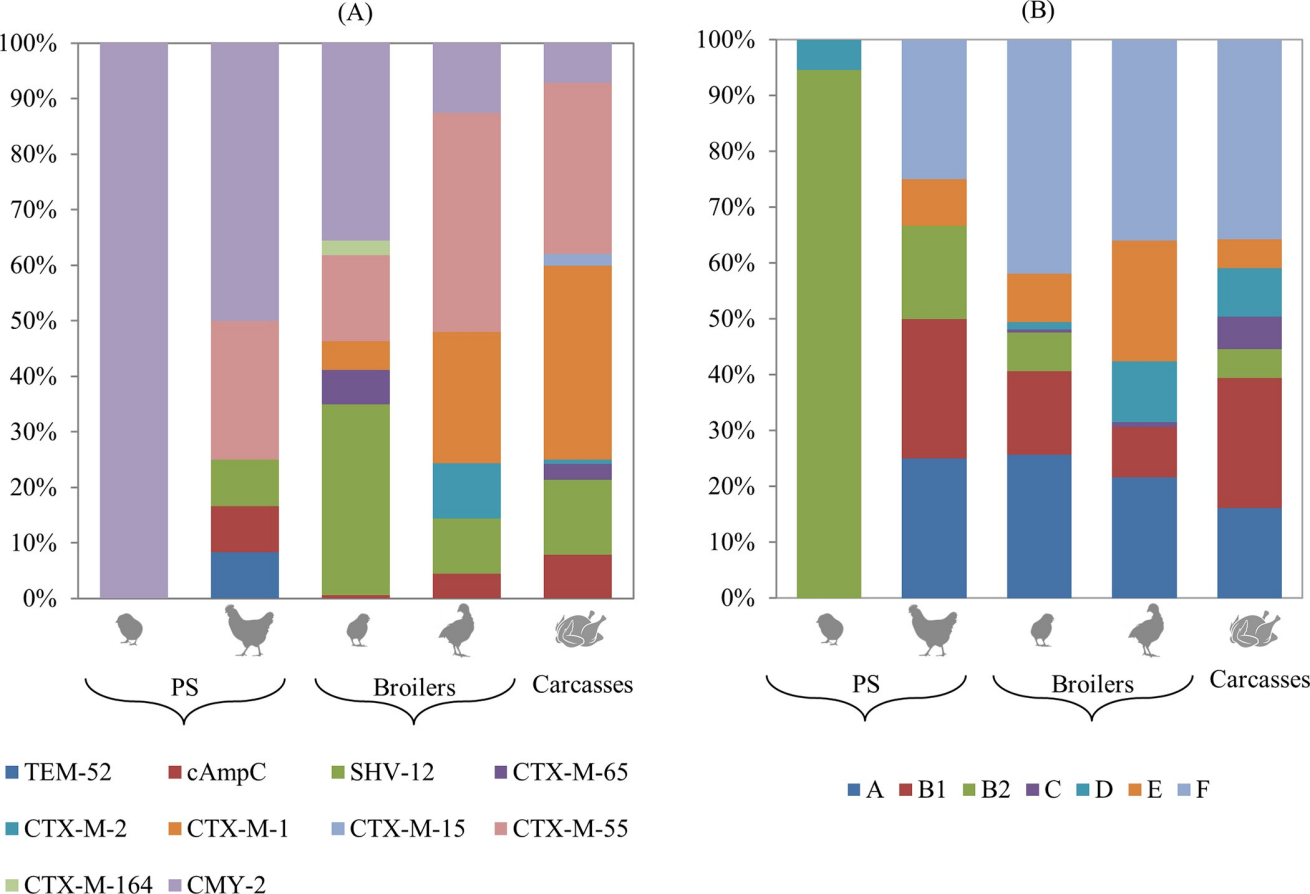
**Distribution of ESBL/pAmpC genes (A) and *E*. *coli* phylogroups (B) in the broiler production pyramid.** (A) cAmpC, isolates with chromosomal mutations in the *ampC* promoter/attenuator. (A) and (B) from left to right: PS chicks, PS breeders, broiler chicks, broilers and carcasses.

Sequencing of ESBL genes revealed variability in *bla*CTX-M-group-1, which was mainly comprised of *bla*_CTX-M-55_ (54.2%) and *bla*_CTX-M-1_ (42.4%), whereas only one isolate (1.7%) was found for each of *bla*_CTX-M-15_ and *bla*_CTX-M-164_ (Fig 2A). Two isolates had concurrent presence of more than one *bla*CTX-M-group-1 genes and thus unassignable by Sanger sequencing. In contrast, all *bla*CMY, *bla*SHV, *bla*TEM belonged to the *bla*_CMY-2_, *bla*_SHV-12_ and *bla*_TEM-52_ variants, respectively. Moreover, the *bla*_CMY-2_ variant was found in all *bla*CMY-carrying isolates.

Overall, the most prevalent gene variants were *bla*_CTX-M-55_ and *bla*_CMY-2_ (27% each) followed by *bla*_CTX-M-1_ (21%) and *bla*_SHV-12_ (14%). Other gene variants were only sporadically present. All (100%) ESBL/pAmpC *E*. *coli* in PS chicks carried *bla*_CMY-2_ (Fig 2A). The relative frequency of this latter gene variant showed a statistically significant decreasing trend (linear slope = -0.20, SE = 0.017, p<0.0001) over subsequent production stages, but was present throughout the production pyramid. *bla*_CTX-M-55_ appeared in PS breeders, remained present in fattening broilers where it peaked at the end of the production cycle (39.6%), and was the second most prevalent gene variant in chicken carcasses (30.7%), after *bla*_CTX-M-1_ (35%). The latter was also present in broiler, but not in breeder flocks (Fig 2A). Both *bla*_CTX-M-1_ and *bla*_CTX-M-55_ showed a significantly increasing trend (linear slope = 0.11, SE = 0.015, p<0.0001, and linear slope = 0.08, SE = 0.017, p<0.0001, respectively) from breeders to carcasses. Furthermore, *bla*_SHV-12_-carrying *E*. *coli* were isolated from all production stages but PS chicks, and were predominantly isolated from fattening broiler chicks (34.6%).

The most frequent *E*. *coli* genotype was phylogroup F (34.9%) followed by A (19.7%), B1 (15.1%) and B2 (12.1%). Phylogroups C, D, and E were each found in less than 10% of isolates. In PS chicks, phylogroup B2 predominated (94.6%), whereas in PS breeders phylogroups F, A, and B1 were equally present (25%) (Fig 2B). In subsequent production levels, phylogroup F was the most prevalent, but other phylogroups were present as well (Fig 2B). Significantly increasing and decreasing trends along the production chain were respectively found for phylogroups F (linear slope = 0.05, SE = 0.01, p = 0.009) and B2 (linear slope = -0.15, SE = 0.01, p<0.0001).

**Fig 3:**
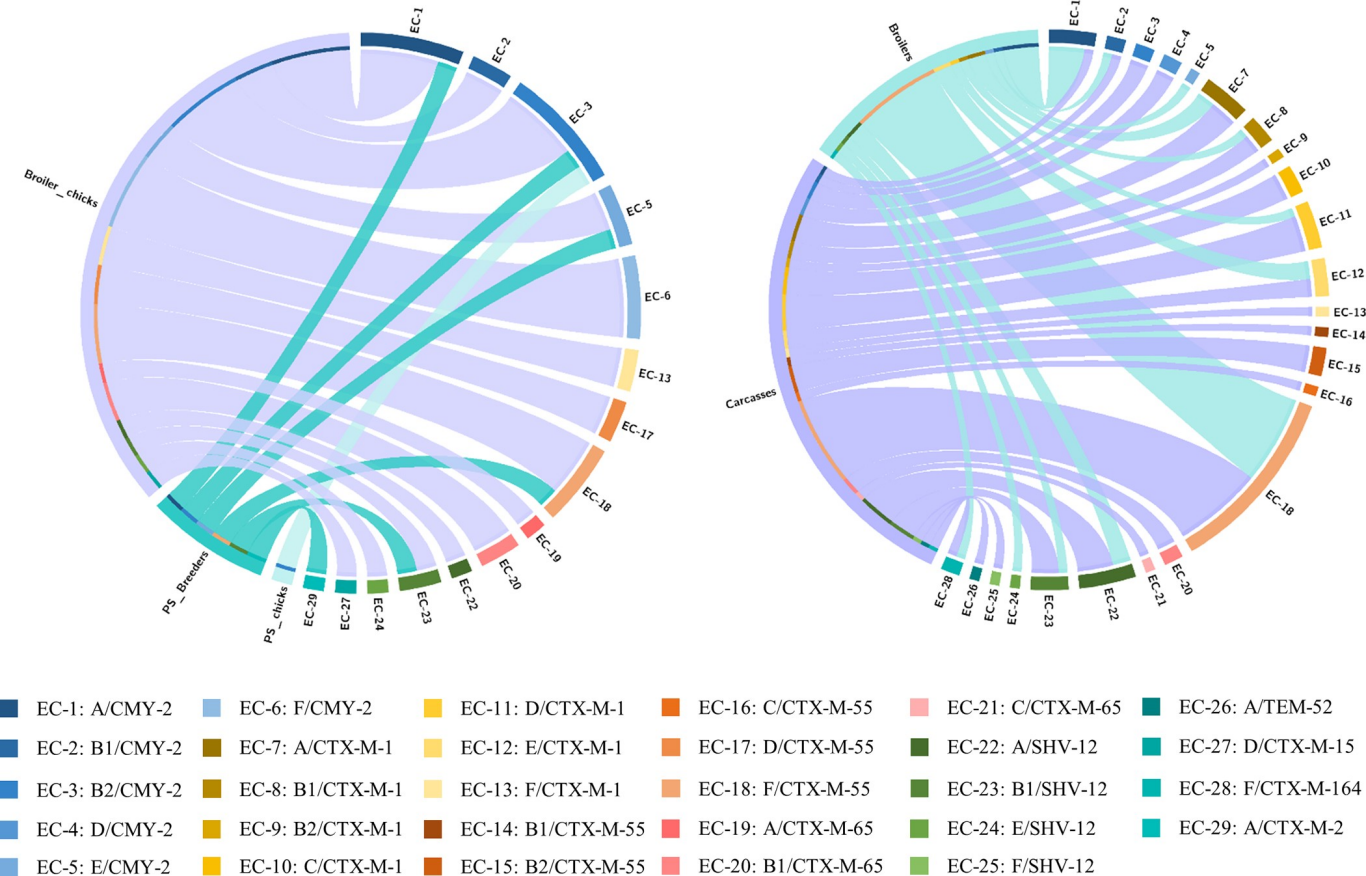
Detected isolates by phylogroup/*bla*-gene combination and corresponding production stages. Size of segments on the right represent the number of isolates with a specific combination. Size of segments on the left represent the number of isolates detected in different production stages. Ribbons connecting left and right segments represent the number of isolates with a specific combination found on the respective production stage. Chord diagram generated with CIRCOS [29].

ESBL/pAmpC genes were found in multiple *E*. *coli* phylogroups within the same production stage, but within the same sampling stage as well (Fig 3). However, phylogroup F-*bla*_CTX-M-55_ was the most frequent (21.1%) isolate profile, especially in fattened broilers (37%) and carcasses (20%). In contrast, phylogroup A- and B2-*bla*_CMY-2_ were the most frequent ones in PS chicks (100%) and PS breeders (33.4%) (Fig 3).

### ESBL/pAmpC *E*. *coli* transfer across the production pyramid

**Fig 4:**
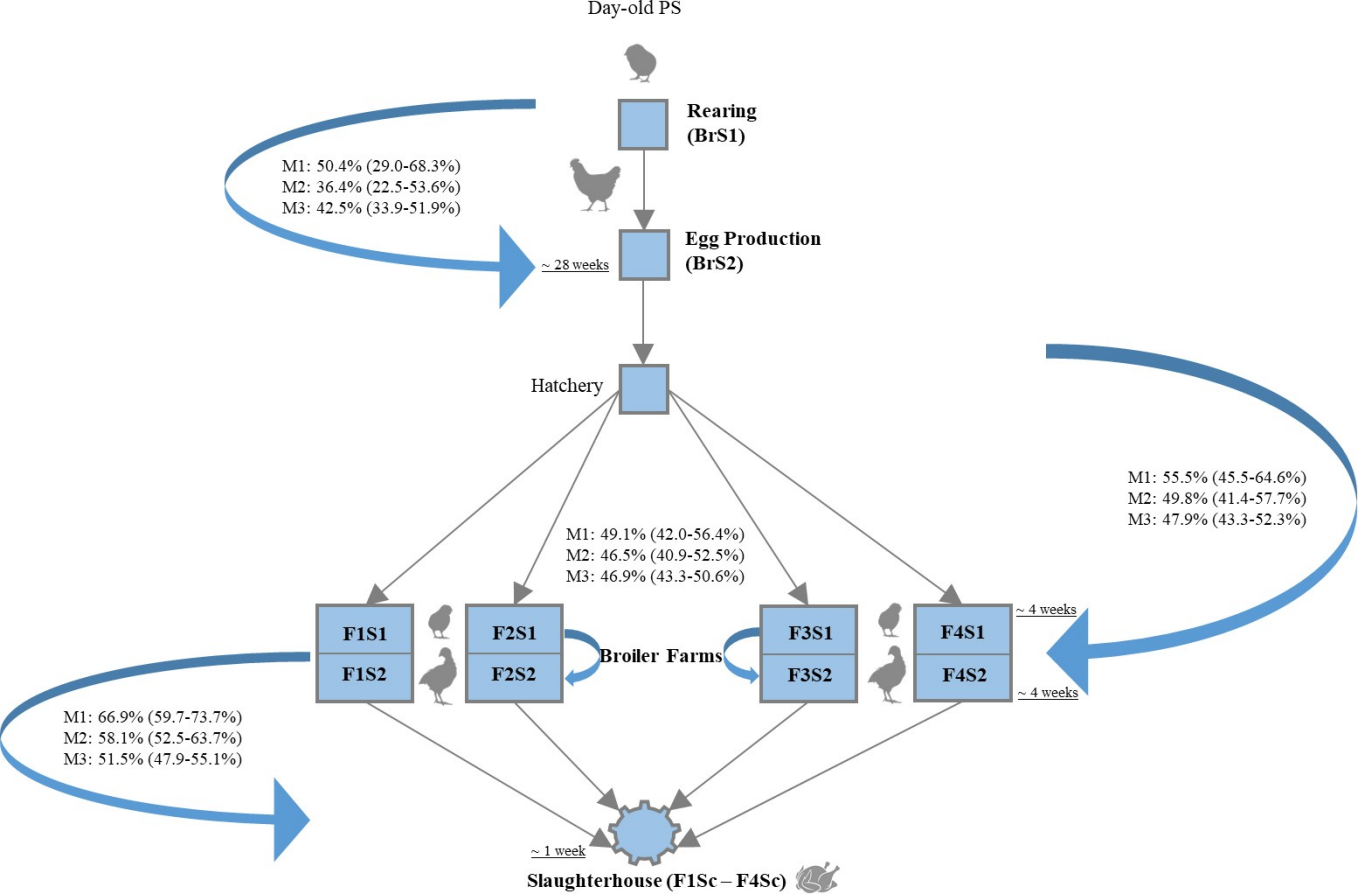
Transfer of ESBL/pAmpC *E*. *coli* across the production pyramid. BrS1, PS chicks; BrS2, PS breeders; F1-4S1, farm 1–4 broiler chicks; F1-4S2, farm 1–4 broilers; F1-4Sc, farm 1–4 carcasses. Arrows and text boxes indicate the overlap of genotyping data in subsequent sampling stages (bold) at three levels (M1-M3) of increasing stratification. Underlined text indicates the time elapsed between sampling two consecutive stages.

Genotyping data were used to calculate PSIs over subsequent sampling stages at three levels of increasing stratification (M1, M2, and M3). The average stepwise transfer of ESBL/pAmpC *E*. *coli* over the whole production pyramid was 55.5% (95%CI 48.3–61.3%) for M1, 47.7% (95%CI 42.3–53.4%) for M2, 47.2% (95%CI 44.2–50.3%) for M3. At the top of the production pyramid, 36.4 to 50.4% (depending on the level of genotyping data stratification) of ESBL/pAmpC *E*. *coli* found in PS breeders overlapped with (and could therefore be estimated to originate from) PS chicks (Fig 4). The overlap between fattening chicks and fattened broilers varied from 46.5 to 49.1% (M3 to M1), whereas 51.5 to 66.9% (M3 to M1) of ESBL/pAmpC *E*. *coli* found in carcasses were likely to originate from broilers sampled before slaughter (Fig 4). In general, the overlap of genotyping data decreased with increasing stratification of these data due to decreased chance of finding one-to-one matches of the same genotype between two sampling stages. Moreover, the overlap increased with decreasing time elapsed between two sampling stages (Fig 4).

## Discussion

This is the first study investigating the occurrence and potential transfer of ESBL/pAmpC *E*. *coli* across the whole broiler production pyramid in Italy. Samples predominantly originated from one Italian region, which is however among the most densely populated poultry areas in the country and thus can be considered representative of a large fraction of Italy’s poultry production [30]. Analysis of 820 samples from three broiler production chains resulted in the identification of ESBL/pAmpC *E*. *coli* in all farms and batches of carcasses, with an overall prevalence of 60.3%. Day-old PS chicks showed the highest prevalence, comparable with recent findings from the Netherlands [31], but higher than those found in Norway [7]. All ESBL/pAmpC *E*. *coli* found in PS chicks had the AmpC phenotype, carried *bla*_CMY-2_, and belonged predominantly to phylogroup B2. Therefore, a prevalent *E*. *coli bla*_CMY-2_ lineage seems to have been introduced through the import of day-old PS chicks. However, due to the widespread occurrence of *bla*_CMY-2_ in poultry, other sources of entry cannot be excluded [32]. It has been speculated that the underlying reason for high prevalence at this production level is the *in ovo* use of cephalosporins in supplying (grand)parent hatcheries, which selects for ESC-resistant *E*. *coli* in the gut of young hatchlings [8]. This is a problem for Nordic countries supplied by the same breeding stock, as the *bla*_CMY-2_ genotype introduced at the top of the production pyramid in these countries spreads clonally to the bottom, is often the only identified ESBL/pAmpC gene [7,33,34]. Similar findings have been described in Denmark [12]. In contrast, we found a significant decrease of *bla*_CMY-2_ and a gradual substitution by mainly *bla*_CTX-M-55_ and *bla*_CTX-M-1_ along the production pyramid, as well as the sharp reduction of prevalence in PS breeders (Fig 1). A similar decrease in the prevalence of *bla*_CMY-2_-carrying *E*. *coli* from 95% in week 1 to 0% in week 21 has been described by Dame-Korevaar *et al*. [31] and was attributed to a selective disadvantage of *bla*_CMY-2_-carrying plasmids. Although different EBSL/pAmpC genotypes were identified during the laying period, *bla*_CMY-2_ was still dominant and the overlap of genotype distributions between the two sampling stages in breeders was substantial despite the relatively long period between them (36.4–50.4%). Persistence of *bla*_CMY-2_ has been previously associated with the IncK and IncI1 plasmid types [11].

In broilers at the start of the cycle, 47.9–55.5% of the ESBL/pAmpC *E*. *coli* were likely to have been vertically transmitted from their parents. The impact of the hatchery on colonisation of hatchlings with ESC-resistant *E*. *coli* was not in the scope of this study. However, the role of the hatchery has proven to be trivial since ESBLs/pAmpCs found in the hatchery’s environment (*e*.*g*. dust and incubators) were different than those recovered from the egg surface and hatchlings [20,35]. As Projahn *et al*. [14] showed, true vertical transmission (infection during egg formation) is rare and hatchlings seem to get colonised through a mode of pseudo-vertical transmission with contaminated eggs that enter the hatchery. Nonetheless, the finding that at least 43.3% of ESBLs/pAmpCs recovered in broiler chicks originated from their parents supports the notion of a combination of vertical transmission and environmental contamination from the farm environment, together with rapid proliferation of resistant bacteria, resulting into high prevalence (69.2%) and diversity of ESBL/pAmpC *E*. *coli* in young broilers [36]. The number of positive faecal samples was 15% lower in fattened broilers, which can be explained by the development of immunity to certain (resistant) *E*. *coli* genotypes [37]. However, high transmission ratios and repeated shifts in phylogroups of ESBL/pAmpC *E*. *coli* during the fattening period, such as the ones observed in our study (Fig 3) lead to persistence of ESC-resistant *E*. *coli* in broiler flocks during the fattening period [37,38]. The overall transfer of EBSL/pAmpC-EC from the start to the end of the broiler production cycle was, on average, 46.5–49.1%, which indicates substantial influence of other sources, such as the environment and the effect of previously fattened flocks, on the genotype landscape of ESBL/pAmpC *E*. *coli* [15]. This has been documented by, *e*.*g*., the finding of *bla*_CTX-M-2_ being only present in fattened broilers, but not in fattening chicks (Fig 2A). The high levels of resistance found are difficult to explain since no cephalosporins were administered in the investigated flocks, but the use of amoxicillin, which was frequent in our study (S1 Fig), could have selected for ESBL/pAmpC *E*. *coli* [7].

At the end of the production pyramid, a large proportion of carcasses (61.3%) were found to be contaminated with ESBL/pAmpC *E*. *coli*, which is comparable with prevalence found in previous studies [16,39,40], although the load of resistant *E*. *coli* was lower in our study. This is a worrying finding since contamination of meat with ESBL/pAmpC *E*. *coli* is directly linked with human exposure [21]. The contribution of chicken meat as a source of human ESBL-pAmpC *E*. *coli* infections is debatable [19,20,41], however, the finding of phylogenetically related strains with the same gene and plasmid profile shared between patients and poultry meat [17,42–44] warrants for measures to prevent the entrance of ESBL/pAmpC *E*. *coli* in the lower levels of the food chain. The highest percentage of transfer of ESBL/pAmpC *E*. *coli* between fattened broilers and carcasses points to contamination of carcasses during processing at the slaughterhouse [39], but the time elapsed between these two sampling stages was the shortest (Fig 4). However, results at this production stage need to be interpreted with caution as cross-contamination from previously slaughtered flocks via other routes (*e*.*g*. scalding water, defeathering machines, transportation crates) is frequent [16,39,45].

Our study proved that the genetic background of ESBL/pAmpC *E*. *coli* in broilers is complex in all production stages, except perhaps for PS chicks, which isolates belonged predominantly to phylogroup B2-*bla*_CMY-2_ (Figs 2 and 3). In subsequent production stages, we found common (*e*.*g*. *bla*_CTX-M-1_, *bla*_SHV-12_) and less common (*e*.*g*. *bla*_CTX-M-65_, *bla*_CTX-M-2_) poultry-related ESBL/AmpC genes [8] to be associated with more than one phylogroup (Fig 3), even within the same farm (data not shown), suggesting that horizontal gene transfer contributes to the dissemination of resistance genes, as previously discussed [13]. In contrast, persistence of phylogroup F-*bla*_CTX-M-55_ in significant proportions of isolates from breeders to the slaughterhouse is indicative of clonal transmission (Fig 3). An unexpected discovery was the occurrence of *bla*_CTX-M-55_ in all production stages except for PS chicks, especially in fattened broilers where it was the most dominant gene (Fig 2A). This is an ESBL gene usually found in food-producing animals and humans in Asia [46] and has been rarely described in poultry in Europe [18,32]. Regarding *E*. *coli* genotypes, from PS chicks we observed a significant reduction of the predominant phylogroup B2, which was mainly substituted by group F in broilers and carcasses (Fig 2B). Both phylogroups are associated with extra-intestinal pathogenicity, as they often harbour virulence genes not found in other *E*. *coli* genotypes [47]. However, isolates belonging to commensal groups (A, B1, and C) were substantially present as well (37% of all isolates) (Fig 2B).

In conclusion, ESBL/pAmpC *E*. *coli* were detectable at all levels of the broiler production pyramid. The highest prevalence was observed in imported PS chicks, with almost all samples being positive for ESBL/pAmpC *E*. *coli*, particularly to *bla*_CMY-2_. Measures to ensure ESBL-free pedigree flocks in supplying countries and avoid introduction of resistant clones by the purchase of (grand)parent stock is therefore warranted [12,18]. Based on the similarity of ESBL/pAmpC *E*. *coli* genotype distributions between subsequent production levels, we showed that the transfer of ESBL/pAmpC *E*. *coli* across the whole production pyramid is likely to be substantial, with approximately half of the genotypes found in a given production stage being likely to originate from the previous stage, and with the time between sampling stages and discriminatory power of genotyping data having an effect as well. Interventions like competitive exclusion [38] may help to control dissemination of ESBL/pAmpC *E*. *coli*. Mitigation strategies should definitely include biosecurity and disinfection measures to avoid colonisation from environmental sources [15,21]. Prevention of cross-contamination in the slaughterhouse processing line is also crucial, as the load of ESBL/pAmpC *E*. *coli* on meat defines human exposure.

## Supporting information

S1 Table Information on the three sampled broiler production chains. (DOCX)

S1 Fig Hierarchical structure of sampled farms. BrS1, PS chicks; BrS2, PS breeders; F1-4S1, farm 1–4 broiler chicks; F1-4S2, farm 1–4 broilers; F1-4Sc, farm 1–4 carcasses. amx, amoxicillin; enr, enrofloxacin; oxtet, oxytetracycline; dox, doxycycline. Samples were not collected from PS chicks of chain B.
(TIF)

S1 File Raw data file. Excel file containing the raw data of this study.
(XLSX)
