# Supplementary material for: Assessing the occurrence and transfer dynamics of ESBL/pAmpC-producing Escherichia coli across the broiler production pyramid
Source: PLoS One. 2019 May 17;14(5):e0217174. doi: 10.1371/journal.pone.0217174 (PMC6524947; doi:10.1371/journal.pone.0217174)
Supplement: S1 Table — (DOCX) [file pone.0217174.s001.docx]

S1 Table. Information on the three sampled broiler production chains.

| Chain | Production stage ^a^ | Date of sampling | Age at sampling ^b^ |
| --- | --- | --- | --- |
| A | PS chicks - PS breeders | 05/01/2017 - 04/07/2017 | 1d - 26w |
|  | F1_broiler chicks - F1_broilers - F1_carcasses | 21/07/2017 - 23/08/2017 - 31/08/2017 | 1d - 33d |
|  | F2_broiler chicks - F2_broilers - F2_carcasses | 28/07/2017 - 29/08/2017 - 04/09/2017 | 1d - 32d |
|  | F3_broiler chicks - F3_broilers - F3_carcasses | 03/08/2017 - 06/09/2017 - 07/08/2017 | 1d - 35d |
|  | F4_broiler chicks - F4_broilers - F4_carcasses | 16/08/2017 - 14/09/2017 - 18/09/2017 | 2d - 33d |
| B | PS breeders | 10/01/2017 | 29w |
|  | F1_broiler chicks - F1_broilers - F1_carcasses | 08/02/2017 - 06/03/2017 - 16/03/2017 | 1d - 28d |
|  | F2_broiler chicks - F2_broilers - F2_carcasses | 08/02/2017 - 06/03/2017 - 16/03/2017 | 1d - 28d |
|  | F3_broiler chicks - F3_broilers - F3_carcasses | 22/02/2017 -20/03/2017 - 28/03/2017 | 2d -28d |
|  | F4_broiler chicks - F4_broilers - F4_carcasses | 22/02/2017 - 20/03/2017 - 29/03/2017 | 2d -28d |
| C | PS chicks - PS breeders | 19/04/2017 -15/11/2017 | 1d - 30w |
|  | F1_broiler chicks - F1_broilers - F1_carcasses | 05/12/2017 - 03/01/2018 - 09/01/2018 | 1d - 28d |
|  | F2_broiler chicks - F2_broilers - F2_carcasses | 12/12/2017 - 09/01/2018 - 17/01/2018 | 1d - 27d |
|  | F3_broiler chicks - F3_broilers - F3_carcasses | 18/12/2017 - 16/01/2018 - 24/01/2018 | 1d - 30d |
|  | F4_broiler chicks - F4_broilers - F4_carcasses | 19/12/2017 - 16/01/2018 -24/01/2018 | 1d - 29d |

^a^ F1-F4, Farm 1-4.

^b^ w, weeks; d, days.
