# Supplementary figures and images for: Assessing the occurrence and transfer dynamics of ESBL/pAmpC-producing Escherichia coli across the broiler production pyramid
Source: PLoS One. 2019 May 17;14(5):e0217174. doi: 10.1371/journal.pone.0217174 (PMC6524947; doi:10.1371/journal.pone.0217174)

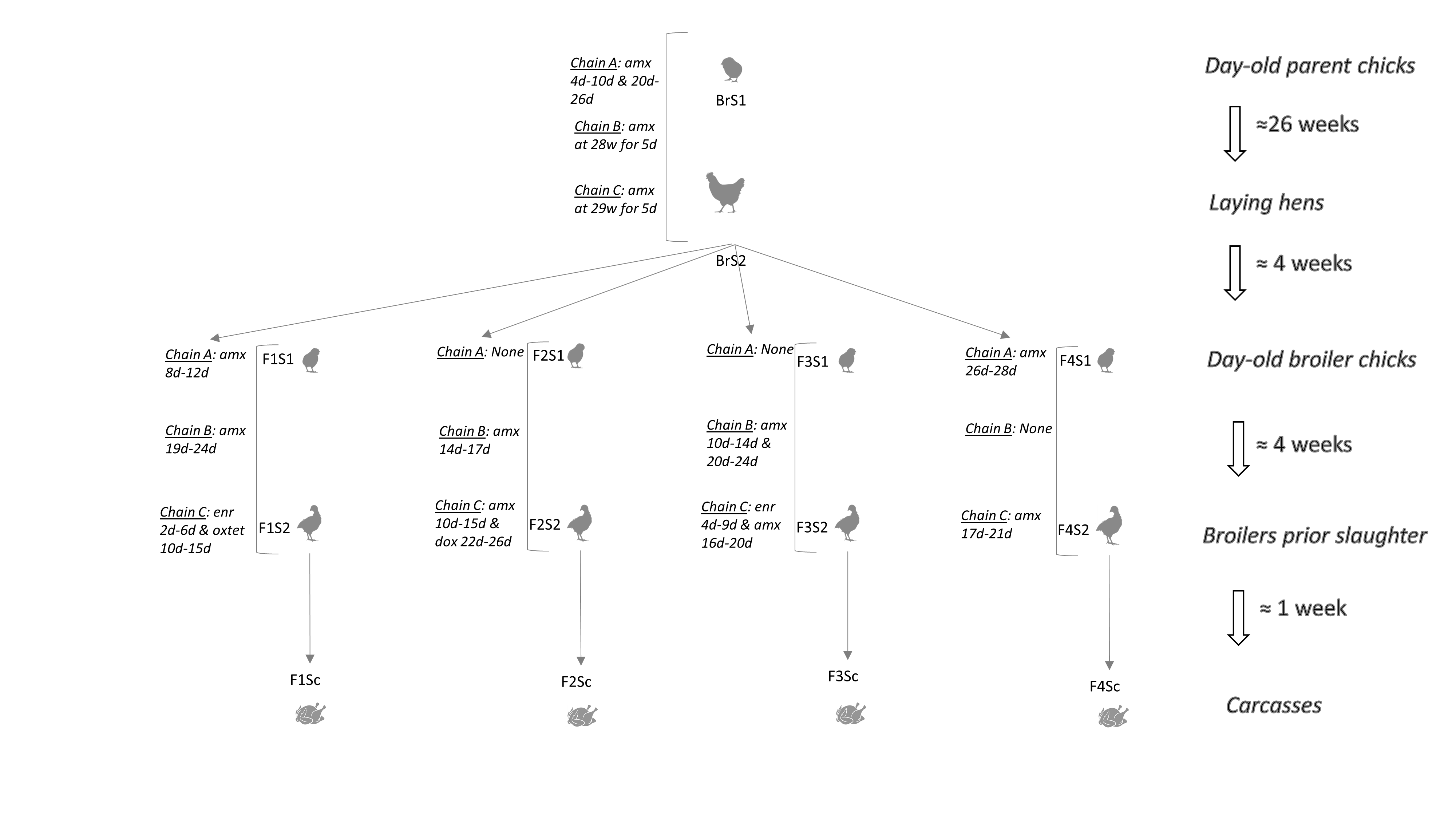

Supplement: S1 Fig — BrS1, PS chicks; BrS2, PS breeders; F1-4S1, farm 1–4 broiler chicks; F1-4S2, farm 1–4 broilers; F1-4Sc, farm 1–4 carcasses. amx, amoxicillin; enr, enrofloxacin; oxtet, oxytetracycline; dox, doxycycline. Samples were not collected from PS chicks of chain B. (TIF) [file pone.0217174.s002.tif]
